# Supplementary figures and images for: Destruction of ERP responses to deviance in an auditory oddball paradigm in amyloid infusion mice with memory deficits
Source: PLoS One. 2020 Mar 11;15(3):e0230277. doi: 10.1371/journal.pone.0230277 (PMC7065782; doi:10.1371/journal.pone.0230277)

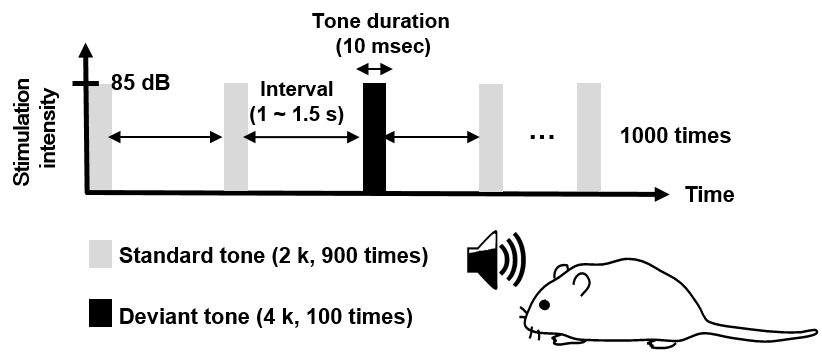

Supplement: S1 Fig — Sound stimuli for the auditory oddball test were described in diagrammatic depiction. Deviant (black) and standard (gray) tones were randomly presented for 10 ms with 1:9 ratio through the speakers around mice. Interstimulus intervals randomly changed in the range from 1 to 1.5 s. (TIF) [file pone.0230277.s001.tif]

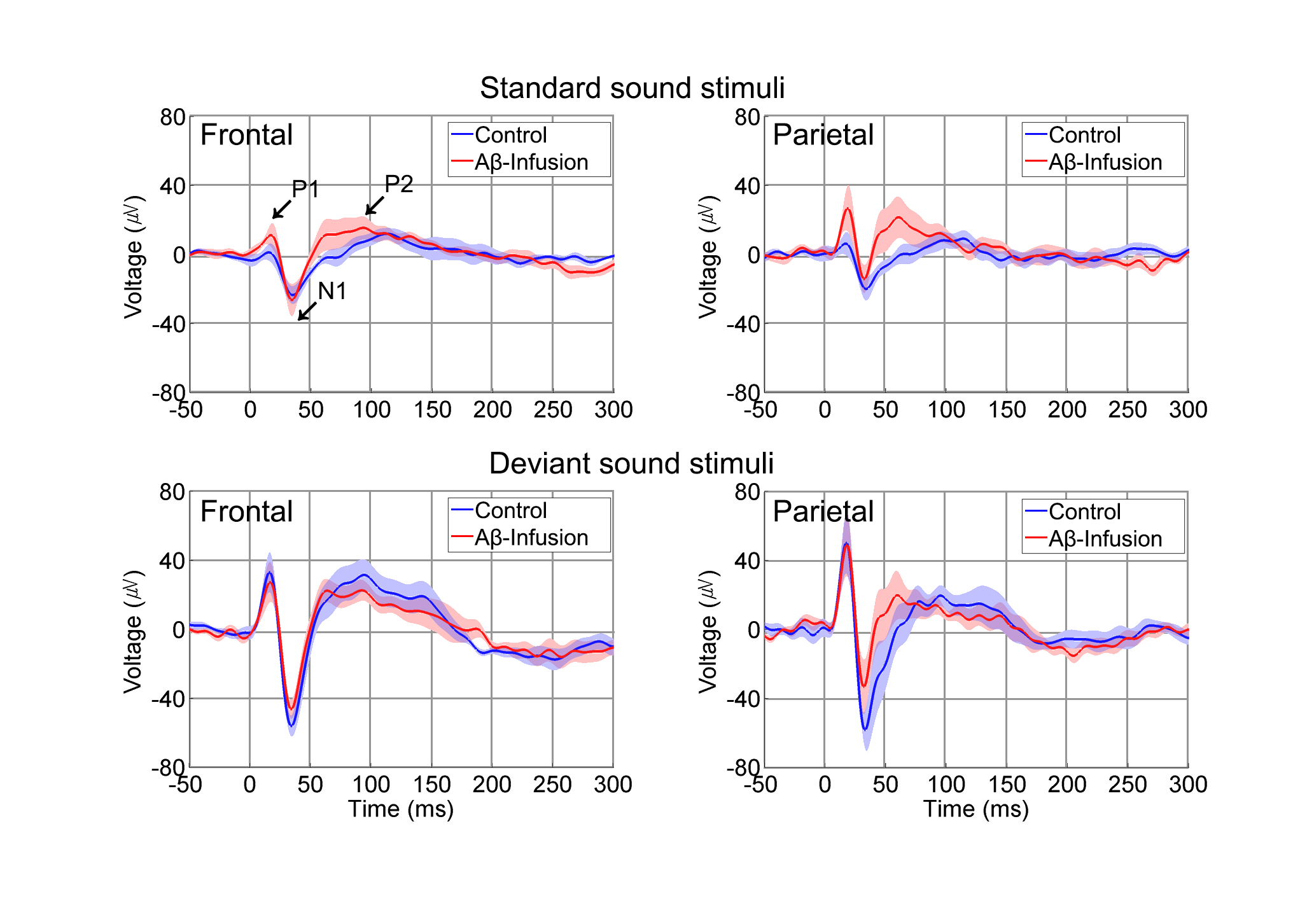

Supplement: S2 Fig — Grand-averaged ERP traces for control (blue-line) and Aβ-infusion (red-line) group were compared. ERP waveforms elicited by standard (top) and deviant (bottom) tones in frontal (left) and parietal (right) regions were presented. Sounds were presented at time zero. In the top-left panel, arrowheads pointed the P1, N1 and P2 components. A significant difference in ERP time trace between control and Aβ-infusion groups were not detected (Student’s t-test, p<0.05). (TIF) [file pone.0230277.s002.tif]
