# Supplementary material for: Destruction of ERP responses to deviance in an auditory oddball paradigm in amyloid infusion mice with memory deficits
Source: PLoS One. 2020 Mar 11;15(3):e0230277. doi: 10.1371/journal.pone.0230277 (PMC7065782; doi:10.1371/journal.pone.0230277)
Supplement: Attachment — Submitted filename: PLoSONE_abeta_rev_letter_fin.docx [file pone.0230277.s003.docx]

Here, we provide a detailed point-by-point response to the reviewers’ comments below. The font styles are following:

- *Reviewer’s comments are in black italic.*
- Authors’ responses are in blue.
- Sentences from original manuscript are in gray highlighted.
- Sentences from revised manuscript are in yellow highlighted.

We hope our rework meets the requirements

*Reviewer #1: The authors conducted an auditory oddball test on the Alzheimer’s disease mouse model with infusing amyloid-beta intra-ventricle. They present data showing amyloid beta eliminated the difference of ERP signature, and showing the difference of MMN in A-beta group for the first time. They suggest this study may provide a markers of sensory memory in mouse AD model.
This manuscript describes a technically sound data and the experiments conducted rigorously. The conclusion is well drawn from data.
This manuscript can be re-evaluate to publish after some minor revisions performed.
Minor revision*

We appreciate your corrections. We corrected the errors as below.

*1. affiliation has typo.*

We deleted the typo.

^2^Division of Bio-Medical Science & Technology, KIST School, Korea University of Science and Technology, Seoul, Republic of Koreaparentheses

^2^Division of Bio-Medical Science & Technology, KIST School, Korea University of Science and Technology, Seoul, Republic of Korea

*2. line7, ‘Alzheimer’s mouse model’ may change to “Alzheimer’s disease mouse model”*

We changed the sentence as you suggested.

an Aβ-infused Alzheimer’s mouse model

an Aβ-infused Alzheimer’s disease mouse model

*3. line 138, “method3” may be typo.*

It was a style error for referencing. We corrected the error.

method3 was changed to

method [4].

*4. line 204, “studies27” may be type.*

studies27 was changed to

studies [27]

*5. line 210, character “beta” is missing*
We fixed the error.

Aβ- was changed to Aβ-

*Reviewer #2: The manuscript reports an interesting study of an Aβ infusion mouse model of Alzheimers Disease (AD). They investigate behavioural measures of spatial memory using the Y-maze task and electrophysiological measures of sensory memory using the oddball paradigm comparing deviant and standard responses. They report impaired performance of the AD mouse model on the Y- maze task as well as evidence that deviant responses in the oddball paradigm and perhaps mismatch like response are also reduced in the AD mice compared with the control mice.*

*1. The manuscript is clearly written, however this could be developed further in particular to tie together the interaction of the abeta oligomers with the likely interaction point at the level of the neuron that is then assayed by the electrophysiological tests – and how this correlates with assaying.*

[Reply] We agree with the reviewer’s opinion of adding value by collimating the pathological and electrophysiological changes by Aβ infusion. Since the pathological data can be obtained after the experiment, it would be desirable to record EEG as close as possible to the injection time point. But the 4-week delay in EEG recording was inevitable to reach sufficient mouse welfare and health conditions required for obtaining reliable EEG responses. For instance, differential ERP is known to be reduced in inattentive or fatigued or unhealthy animals. Hence, we endowed animals 3 weeks before implantation surgery and another week before recording, for full recovery from infusion and implantation surgery, respectively. We believe that the Aβ oligomer injection influences the central nervous system in a devastating way. It is known that Aβ infusion induces an irreversible change in brain tissue: Cardinal features of Aβ infusion include synapse loss, tau hyperphosphorylation, astrocyte and microglial activation, and most importantly, neurofibrillary tangle formation, which is irreversible (Forny-Germano et al., J Neurosci, 2014).

Our study shows an irreversible influence of ICV injection fingerprinted in the ERP results, which has a translational value because of its availability for cross-species comparison between human patients and the mouse model.

*2. Was any work done to corroborate the distribution of the Abeta oligomers are ICV (whether within this study of previously)?*
[Reply] We have reported a brain section-level evidence of Aβ infusion in brain tissue in our previous methodology paper (Kim et al., J. Vis. Exp., 2016). However, the propagation of infusion effect over time through various brain regions has not been studied yet. We expect that the devastating effect of Aβ infusion will not be restricted to the injection site since it affected differential ERP, engaging both bottom-up and top-down cortical circuits.

*3. How do the concentrations and timings and electrophysiological findings correlate or differ from work done using abeta oligomer incubations in brain slice (rodent), or biopsy derived human material? This is important in the overall experimental rationale.*

[Reply] We appreciate this comment. As far as we know, a direct comparison between biopsy-derived human material and brain slice of infusion model has not yet been reported. Notwithstanding, the Aβ-ICV injection model has been widely investigated with variations of Aβ aggregate species, Aβ isomers, injection numbers, and base animal models accompanied by behavior tests. However, it is hard to find complete datasets of pathology and behaviors in terms of timing and concentration. Nonetheless, several research articles (cited in our manuscript) used similar experimental conditions to ours (~ 2 ug concentration, one-month delay after injection in observation). Here are the corresponding papers.

- Scientific Reports (2015) 5, 11708 reports that memory deficits and synaptoxicity of mice became gradually worse over 3 and 40 days since the single injection of Aβ(1-42) 3 ug.
- Journal of neuroscience research (2010) 88, 2923-2932 reports long-term potentiation changes with fear conditioning and CaN activity results by CV injection of Aβ oligomers in lower amounts than those of our experimental design.
- Neuroscience letter (1994) 170, 63-66 reports behavioral alterations in water-maze by varied concentrations of Aβ monomers in lower amounts than those of our experimental design.

**Reference**

Kim, H. Y., Lee, D. K., Chung, B. R., Kim, H. V. & Kim, Y. Intracerebroventricular Injection of Amyloid-beta Peptides in Normal Mice to Acutely Induce Alzheimer-like Cognitive Deficits. J. Vis. Exp. (109), e53308, 10.3791/53308 (2016).

Forny-Germano, L., e Silva, N. M. L., Batista, A. F., Brito-Moreira, J., Gralle, M., Boehnke, S. E., ... & Klein, W. L. Alzheimer's disease-like pathology induced by amyloid-β oligomers in nonhuman primates. *Journal of Neuroscience*, *34*(41), 13629-13643 (2014).

Ali, T., Yoon, G. H., Shah, S. A., Lee, H. Y., & Kim, M. O. Osmotin attenuates amyloid beta-induced memory impairment, tau phosphorylation and neurodegeneration in the mouse hippocampus.Scientific reports,5(1), 1-17 (2015).

Dineley, K. T., Kayed, R., Neugebauer, V., Fu, Y., Zhang, W., Reese, L. C., & Taglialatela, G. Amyloid‐β oligomers impair fear conditioned memory in a calcineurin‐dependent fashion in mice.Journal of neuroscience research,88(13), 2923-2932 (2010).

Nitta, A., Itoh, A., Hasegawa, T., & Nabeshima, T. β-Amyloid protein-induced Alzheimer's disease animal model.Neuroscience letters,170(1), 63-66 (1994).
